# Aqueous Synthesis, Degradation, and Encapsulation of Copper Nanowires for Transparent Electrodes

**DOI:** 10.3390/nano8100767

**Published:** 2018-09-28

**Authors:** Josef Mock, Marco Bobinger, Christian Bogner, Paolo Lugli, Markus Becherer

**Affiliations:** 1 Chair of Nanoelectronics, Technical University of Munich, 80333 Munich, Germany; josef.mock@tum.de (J.M.); christian.bogner@tum.de (C.B.); markus.becherer@tum.de (M.B.); 2 Faculty of Science and Technology, Free University of Bolzano, 39100 Bolzano-Bozen, Italy; paolo.lugli@unibz.it

**Keywords:** copper nanowires, CuNWs, degradation, encapsulation, PDMS, PMMA, solution-based, transparent electrode

## Abstract

Copper nanowires (CuNWs) have increasingly become subjected to academic and industrial research, which is attributed to their good performance as a transparent electrode (TE) material that competes with the one of indium tin oxide (ITO). Recently, an environmentally friendly and aqueous synthesis of CuNWs was demonstrated, without the use of hydrazine that is known for its unfavorable properties. In this work, we extend the current knowledge for the aqueous synthesis of CuNWs by studying their up-scaling potential. This potential is an important aspect for the commercialization and further development of CuNW-based devices. Due to the scalability and homogeneity of the deposition process, spray coating was selected to produce films with a low sheet resistance of 7.6 Ω/sq. and an optical transmittance of 77%, at a wavelength of 550 nm. Further, we present a comprehensive investigation of the degradation of CuNWs when subjected to different environmental stresses such as the exposure to ambient air, elevated temperatures, high electrical currents, moisture or ultraviolet (UV) light. For the oxidation process, a model is derived to describe the dependence of the breakdown time with the temperature and the initial resistance. Finally, polymer coatings made of polydimethylsiloxane (PDMS) and polymethylmethacrylate (PMMA), as well as oxide coatings composed of electron beam evaporated silicon dioxide (SiO_2_) and aluminum oxide (Al_2_O_3_) are tested to hinder the oxidation of the CuNW films under current flow.

## 1. Introduction

Transparent electrodes (TEs) are commonly fabricated by materials such as carbon nanotubes (CNTs) [1], poly(3,4-ethylene dioxythiophene) polystyrene sulfonate (PEDOT:PSS) [2], graphene [3], graphene oxide [4], indium tin oxide (ITO) [5] and metal nanowires (MNWs) such as silver nanowires (AgNWs) [6] and copper nanowires (CuNWs) [7]. Hybrid systems composed of a combination of these materials such as CuNWs/graphene oxide [8], AgNWs/PEDOT:PSS [9] and AgNWs/CNTs [10] were also employed for TEs. Recently, transparent metal meshes have attracted some research interest, which is attributed to their low resistance. These meshes are made of (i) printed and electroplated silver and nickel electrodes [11], (ii) transfer printed copper grids [12] as well as (iii) copper electrodes with a micromesh structure that is patterned using ultraviolet (UV) lithography and wet etching [13].

Due to their high electro-optical performance [14], ease-of-processing in great quantities [15,16] as well as facile large-area deposition techniques at ambient conditions such as spray-coating [17], AgNWs and CuNWs are increasingly considered as next generation transparent electrodes that are able to compete with ITO [18]. It should be noted that for metal nanowires, AgNWs are the major competitor for CuNWs. Both types of metal nanowires have advantages and disadvantages. On the one hand, AgNWs possess the advantage of a high chemical robustness [19]. Further, there are rapid and recent advancements in the number and the relevance of the applications for AgNWs [20,21,22,23] as well as the improvement of the film stability [24,25,26], whereas CuNWs readily oxidize above a temperature of 150 °C [27], under ambient conditions. On the other hand, CuNWs are grown from low-cost copper-containing precursor salts. The extremely high abundance of copper on earth compared to silver leads to a copper price that is lowered by around a factor of 80 compared to the price for silver [28,29,30].

Applications for the aforementioned TE materials cover a broad spectrum that ranges from transparent heaters [31], thermoacoustic speakers [32,33], solar cells [34], microlenses [35], transparent antennas [36], photodiodes [37], touch panels [38] and organic light-emitting diodes (OLEDs) [1] to electromagnetic interference shieldings [39], as well as piezo- [40] and pyroelectric [41] energy conversion.

**Figure 1:**
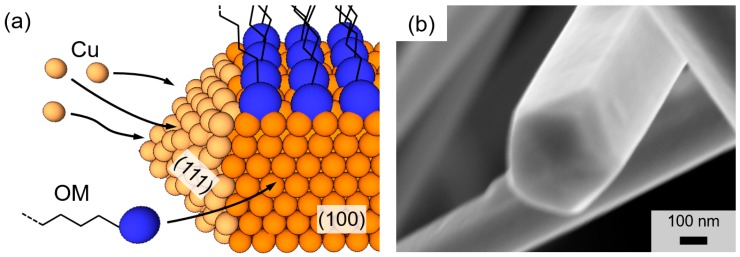
(**a**) Schematic for the growth of a single copper nanowire (CuNW) along the (111)-plane. The blue and orange spheres indicate oleylamine (OM) head groups and copper atoms, respectively. (**b**) High-resolution field-emission scanning electron microscope (FESEM)-image for a single copper nanowire with the characteristic pentagonal shaped cross-section.

The first solution-based synthesis of CuNWs was reported by Chang et al. in 2005 [42]. In their work, copper(II) nitrate Cu(NO_3_)_2_ was utilized as the copper-containing precursor, ethylenediamine (EDA) as the capping agent and hydrazine (N_2_H_2_) was used as a reducing agent for the copper nitrate and the solvent. Since then, numerous studies that are summarized in References [27,43] have been published. A significant advancement towards an environmentally friendly synthesis has been achieved by Hwang et al. in 2016, where DI water instead of hydrazine was employed as the solvent [44]. The synthesis of the CuNWs in this contribution is based on the protocol of Hwang et al. that we recently tailored with regard to the material ratios, the process time and the process temperature, as well as the addition of alcoholic co-solvents [27]. For our synthesis, copper(II) chloride dihydrate (CuCl_2_·2H_2_O), was used as the precursor material and *L*-Ascorbic acid (AA) was used as an environmentally friendly and mild reducing agent. Oleylamine (OM) served as the capping agent and DI water was utilized as the solvent. The self-seeding process requires neutral Cu^0^ atoms, which are provided by the oxidation of AA to dehydroascorbic acid that goes along with the reduction of Cu^2+^-ions to Cu^0^-atoms. As sketched in Figure 1a that depicts a schematic for a single CuNW growth, OM has an increased adsorption rate for the (100) plane compared to the (111) plane [45]. This higher adsorption rate leads to the passivation of the wire shell and in turn, allows for the uniaxial wire growth along the (111) plane [44]. In agreement with the literature [15,44,46], the resulting CuNWs have a face-centered cubic (fcc) structure and a pentagonal cross-section [47,48], as shown in the high-resolution field-emission scanning electron microscope (FESEM) image of a single CuNW in Figure 1b.

It can be concluded that the growth mechanism of CuNWs is well understood and the aqueous synthesis protocol is tailored to an extent that allows producing TEs with properties comparable to the ones of AgNWs and ITO. However, for the commercialization of CuNWs, their up-scaling potential, that has not yet been studied for the aqueous synthesis, is crucial. Thus, in this work, we investigate the effect of the precursor-to-solvent mass ratio on the wire growth. A large drawback of CuNWs that cannot be omitted is their gradual oxidation at ambient conditions, which is readily increased for temperatures above 100 °C [49]. As tested by a few groups with success, this oxidation can be slowed by encapsulating the CuNWs with following approaches: (i) applying polymer coatings composed of polydimethylsiloxane (PDMS) [49], polymethylmethacrylate (PMMA) [49], PEDOT:PSS [50], and polyurethane acrylate resin [51] (ii) atmospheric pressure spatial atomic layer deposition (AP-SALD) of aluminum oxide (Al_2_O_3_) and (iii) the electrodeposition of zinc, tin and indium shells onto the nanowires, followed by their oxidation [30]. Besides encapsulation, there are also works that report on the laser-induced nanowelding of CuNWs [52] and even the photothermochemical reduction [53,54] of oxidized and non-conductive CuNW networks to highly conductive ones. Both the nanowelding of CuNW junctions and the laser-induced reduction of CuNWs improved the oxidation robustness of the films. However, to this day, a comprehensive study for the degradation of CuNWs with regards to various environmental stresses and a model for the oxidation mechanism are still missing.

Therefore, in this work, we report on a rigorous degradation study for CuNW films that are subjected to ambient air, elevated temperatures, high electrical currents, moisture, and UV light. A purposely developed and novel model that describes the time- and temperature- dependent formation of an oxide is presented. Encapsulations made of polymers or ebeam evaporated oxides are deposited to the CuNW films to increase their lifetime. Further, to the best of our knowledge, the scaling potential of the presented aqueous synthesis of CuNWs is for the first time studied by varying the precursor-to-solvent ratio. This study aids in assessing the scaling and in turn also the commercialization potential of the presented synthesis protocol.

## 2. Materials and Methods

### 2.1. Synthesis of Copper Nanowires

The protocol for the aqueous synthesis of CuNWs was adapted from one of our previous publications [27]. For a standard synthesis, 300 mg copper(II) chloride dihydrate (CuCl_2_·2H_2_O) (Sigma-Aldrich, St. Louis, MO, USA, C3279) were immersed in 25 g deionized (DI) water and bath sonicated for a duration of 5 min. Then, 900 mg oleylamine (Sigma-Aldrich, St. Louis, MO, USA, O7805) was added and the solution was horn sonicated for 90 s at a power of 200 W, using an S-450 Digital Sonifier^®^ from Branson Ultrasonics Corporation (St. Louis, MO, USA). Subsequently, 300 mg *L*-Ascorbic acid (Sigma-Aldrich, St. Louis, MO, USA, A92902) dissolved in 5 g DI water was added. The solution was then allowed to age for 12 h in a silicone oil bath at a calibrated temperature of 79 °C. All the reagents were processed without further purification. For the study of the precursor-to-solvent weight ratio, the content of DI water was kept constant and the remaining materials were changed accordingly. 

### 2.2. Synthesis Analysis

Scanning electron microscope (SEM)-images were recorded with an NVision40 FESEM from Carl Zeiss (Oberkochen, Germany) at an acceleration voltage of 7 kV, an extraction voltage of 5 kV and a working distance of 5–6 mm, which was optimized to achieve the best image quality. The wire lengths were evaluated manually using the software Gwyddion (v2.48). For an automated evaluation, DiameterJ (v1.018), a plugin invented for ImageJ (v1.51j8), was utilized. More details on this method are reported elsewhere [28].

### 2.3. Spray Ink Preparation and Deposition

The as-synthesized solution was allowed to cool down to room temperature. The top solution was decanted and the CuNW-containing precipitate was rinsed with isopropyl alcohol (IPA). To remove the OM and eventually a thin oxide shell that has formed after the synthesis, the CuNW precipitate was immersed with 5 wt% propionic acid (Sigma Aldrich, St. Louis, MO, USA, P5561) in 30 g IPA and allowed to react for 5 min. After centrifugation at a speed of 1.75 krpm for a duration of 5 min, the precipitate was immersed in 30 g IPA and sonicated for 20 min in an USC300TH bath sonicator from VWR International (Radnor, PA, USA), at frequency and power of 45 kHz and 80 W, respectively, to improve the dispersion of the CuNWs. As observed in a previous publication, bath sonication with the parameters above for a duration below 30 min does not lead to cracks or deformations of the wires [27]. For the spray deposition, a commercial and handheld airbrush (Triplex II F from Gabbert, Leipzig, Germany) with an orifice of 150 μm was utilized and operated with pressurized nitrogen (1.5 bar). To increase the evaporation of the solvent, the samples were placed on a Thermofisher RT2 hot plate and heated to a temperature of 70 °C. In a glovebox, under nitrogen atmosphere, all CuNW films were subjected to a thermal sintering treatment at a temperature of 200 °C for a duration of 30 min.

### 2.4. Electro-Optical Characterization

The transmittance spectra were recorded in the visible range using a 300 W xenon arc lamp, chopped at a frequency of 210 Hz. The light passes through an Oriel Cornerstone 260 ¼ monochromator and a silicon-based photodiode with a transconductance amplifier that is connected to a 70105 Oriel Merlin digital lock-in amplifier. The calibration of the photodiode was performed with a glass substrate to determine the pure transmission of the CuNW films. The sheet resistances were measured using a four-point probe head from Jandel (Linslade, UK) connected to a B2901A Keysight (Santa Rosa, CA, USA) source measuring unit (SMU). A constant current of 1 mA was sourced for all measurements.

### 2.5. X-ray Photoelectron Spectroscopy

X-ray photoelectron spectroscopy (XPS) measurements were performed at a base pressure of 5 × 10^−10^ mbar using monochromatic *K*_α_ radiation from an aluminium anode that is operated at an electrical input power of 350 W. The spectra were acquired using a SPECS Phoibos hemispherical analyzer at a pass-energy of 30 eV with an energy resolution of 0.05 eV. The raw data were processed using the software CasaXPS from Casa Software Ltd. (Teignmouth, UK). The backgrounds of the spectra were removed by Shirley background subtraction [55].

### 2.6. Degradation Tests

The CuNW films were sprayed to a glass substrate with the dimensions of 5.0 × 5.0 cm^2^ and a thickness of 1.45 mm, in accordance with the process described in Section 2.3 and as reported in a previous work [27]. The films were contacted using commercial copper tape and conductive silver paint RS Pro from RS components (article number: 186-3600, Corby, UK) with a silver content of 50–75%, which resulted in an active CuNW area of 3.5 × 5.0 cm^2^ = 17.5 cm^2^.

#### 2.6.1. Ambient Air

The degradation tests in the ambient air were performed in an air-conditioned lab environment at a temperature and a relative humidity of around 22 °C and 40%RH (=relative humidity), respectively. The samples were shielded from the light to exclude a light-induced degradation that is studied in Section 3.2.5.

#### 2.6.2. Elevated Temperatures

For the degradation with regard to elevated temperatures, the samples were placed on an RT2 hot plate from Thermofisher, under ambient conditions, and heated from room temperature to the desired annealing temperature. The resistance-time curves were tracked in time steps of 1 s using a Keithley 2200-30-5 power supply operated at a small probe current of 5 mA that was sourced to all devices.

#### 2.6.3. Electrical Current

The CuNW films were driven at an electrical and constant DC power of 6 W using a programmable power supply 2200-30-5 from Keithley (Cleveland, OH, USA). To keep the power at a constant level for increasing resistances, a LabVIEW (v2017) program was developed to adjust the sourced current accordingly, after each time step.

#### 2.6.4. Moisture

The CuNW films were placed in a climatic chamber VCL 4006 from Vötsch Industrietechnik GmbH (Balingen, Germany) and the relative humidity was either set to a low value of 20%RH or a high value of 90%RH. For all measurements, the temperature was kept at a constant value of 60 °C and DI water with a resistivity of 18.2 MΩ∙cm was utilized. In accordance with Section 2.6.2. and Section 2.6.3., the resistance-time data was recorded using a programmable power supply, which was operated with a probe current of 5 mA.

#### 2.6.5. UV-Light

UV light illumination of the CuNW films was performed using the illuminator box 1S from Gie-Tec GmbH (Eiterfeld, Germany) equipped with four special fluorescent tubes with a total electrical power consumption of 32 W. According to the manufacturer, the tubes emit in a wavelength range of 350 to 400 nm. The 2probe resistances of the films were measured over a duration of 1 month using a standard handheld multimeter VC830 from Voltcraft (Wollerau, Germany).

## 3. Results

### 3.1. Synthesis of Copper Nanowires

**Figure 2:**
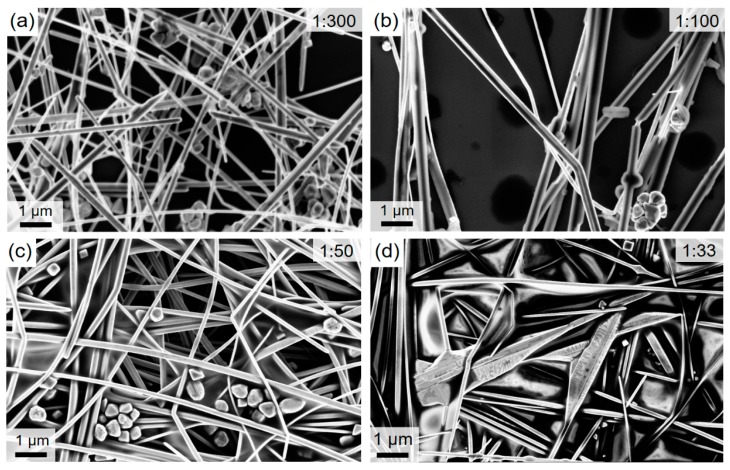
FESEM-images of CuNWs for the precursor-to-solvent series with different mass ratios (precursor:solvent) of (**a**) 1:300, (**b**) 1:100, (**c**) 1:50 and (**d**) 1:33.

**Figure 3:**
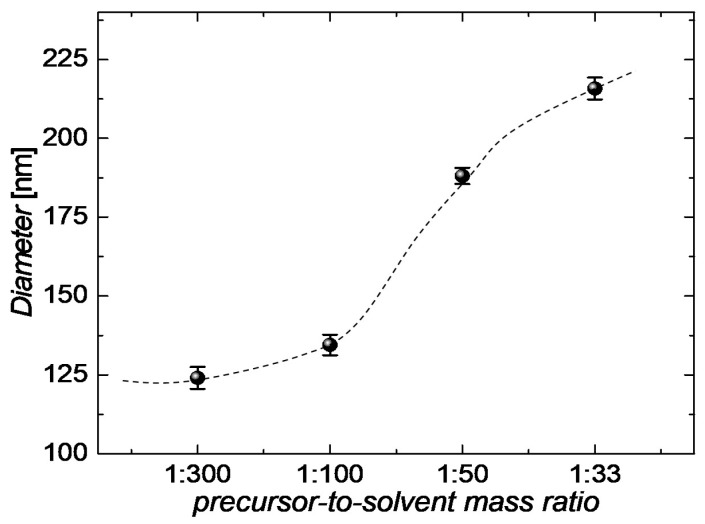
Mean diameters extracted using DiameterJ from the FESEM-images illustrated in Figure A1 for the precursor-to-solvent series with different mass ratios. The dashed line serves as a guide to the eye.

**Figure A1:**
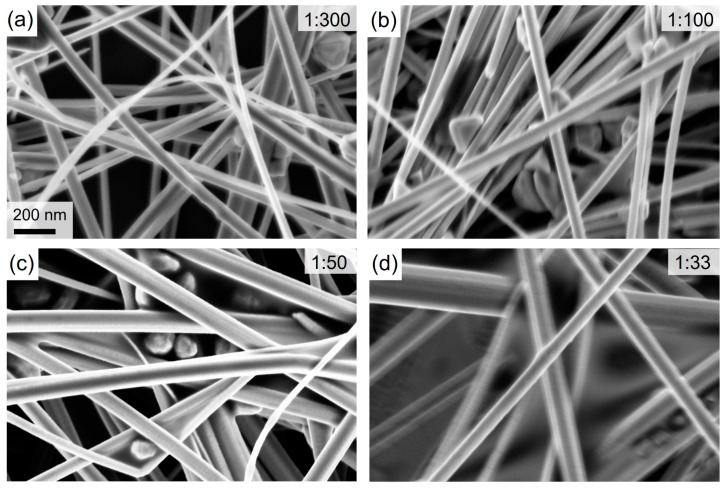
High-resolution FESEM-images of CuNWs for the precursor-to-solvent series with different mass ratios (precursor:solvent) of (**a**) 1:300, (**b**) 1:100, (**c**) 1:50 and (**d**) 1:33. The scale bar in (**a**) applies to all images.

**Figure A2:**
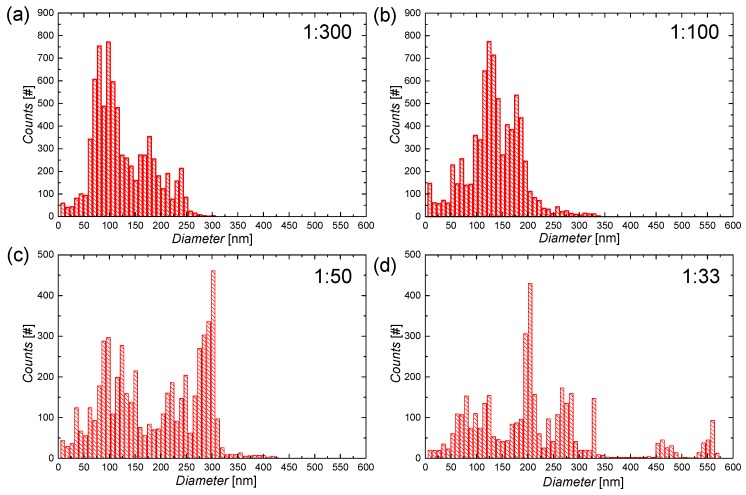
Diameter histograms for the precursor-to-solvent series with different mass ratios (precursor:solvent) of (**a**) 1:300, (**b**) 1:100, (**c**) 1:50 and (**d**) 1:33.

In our previous work, we have tailored the aqueous synthesis of CuNWs [27]. As the next important step that aids to assess the commercialization potential of our synthesis, we report on an up-scaling study for the aforementioned aqueous synthesis of CuNWs. For this, the precursor-to-solvent ratio is varied by around a factor of 10 and the resulting growth product is analyzed with regard to the quality of the dispersion and the nanowire diameters. A low mean diameter for the nanowires is a commonly accepted criterion for a high quality of the synthesis since it is usually accompanied by a high aspect ratio, i.e., length-to-diameter ratio, and a low haze value [56,57]. The SEM-images for the precursor-to-solvent series are depicted in Figure 2 for different mass ratios (precursor:solvent) of (a) 1:300, (b) 1:100, (c) 1:50 and (d) 1:33. Along with the precursor weight, the weights of OM and AA were adjusted accordingly. From the SEM-images in Figure 2c,d for the large precursor contents, following effects can be seen: (i) For the highest ratio of 1:33, the wire morphology is visibly degraded; (ii) the diameters are increased compared to the ones shown in the SEM-images for the lower precursor concentrations in (a) and (b); and (iii) the CuNWs tend to form clusters. After the analysis of the described aqueous CuNW synthesis, a mean diameter of 134 nm ± 4 nm and a mean length of 40 µm ± 21 µm could be obtained for the wires shown in Figure 2b. Employing the method outlined in Section 2.2 and in previous publications [28,29,58], the diameters were analyzed quantitatively and in an automated way from SEM-images (see Appendix A, Figure A1 and Figure A2 for the high-magnification SEM-images and the diameter histograms, respectively). The mean diameters as a function of the precursor-to-solvent ratio are depicted in Figure 3.

From Figure 3 it can be seen that the diameter decreases gradually with a reduction in the precursor concentration from a mean diameter of 215 nm to 124 nm, for a ratio of 1:33 to 1:300, respectively. We believe that there is a critical pre-cursor-to-solvent ratio of around 1:100. Below this ratio, well-defined nanowires with a small diameter grow since the formation of copper-oleylamine micelles is stable. Above a ratio of 1:100, the micelles are not stable enough since the concentrations for copper-oleylamine complexes and subsequently their interaction has increased. Similar to the case of adding alcoholic co-solvents, as previously reported [27,44], the interaction of micelles can lead to their rupture [59,60], which has a negative effect on the uniaxial wire growth since adsorption rates on the (100) and (111) planes are different. In summary, it can be concluded that a range of 10 is sufficient to study the up-scaling potential of the presented synthesis for the following reasons. For a large ratio of 1:33, the wire morphology is clearly degraded, whereas for a low ratio of 1:300, the reduction in diameter is low and already implies a saturation behavior, as can be seen from Figure 3.

### 3.2. Degradation of Copper Nanowires

Up to date, there are only a few studies that report on the degradation of CuNWs. So far, in agreement with the literature, the following stresses have been applied to the CuNW films: exposure to (i) sunlight [61], (ii) elevated temperatures [61], (iii) humidity [61] and (iv) electrical current [49,61]. Nevertheless, a comprehensive study that addresses the major stresses for the chemically-induced degradation of CuNWs is still missing. In the following, the degradation of CuNW films will be studied with regard to (i) exposure to ambient air at ambient temperature, (ii) exposure to elevated temperatures, (iii) prolonged electrical current flow, (iv) different moisture levels and (v) UV-visible light exposure. In Section 3.2.6, for the first time, a model for the temperature-induced oxidation of CuNWs is presented. This model describes the oxide formation on the nanowire shell and relates the breakdown time of the films to the annealing temperature and the initial resistance of the nanowire networks. Further, X-ray photoelectron spectroscopy (XPS) spectra that allow for resolving the chemical alterations induced by the environmental stresses are discussed.

#### 3.2.1. Ambient Air

**Figure 4:**
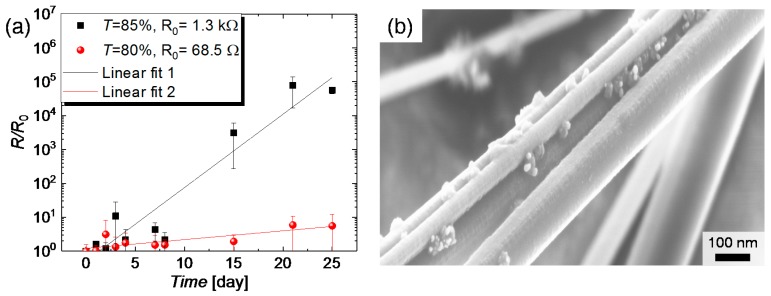
(**a**) Normalized increase in resistance *R*/*R*_0_ of two CuNW films with different transmittances exposed to ambient conditions as a function of the time; (**b**) FESEM-image of CuNWs exposed to ambient conditions for more than 25 days.

A long-term stability test under ambient atmosphere was performed for CuNW films with different initial resistance. Prior to this test and in accordance with Section 2.3, the as-synthesized and spray-deposited CuNW-films were subjected to thermal annealing at a temperature of 200 °C for a duration of 30 min, under a nitrogen atmosphere. It should be noted that this post-deposition treatment was applied for all CuNW films to lower their resistances uniformly across the film and to form a good mechanical and electrical contact at the wire-to-wire junctions, which can aid to improve the robustness of the films. The normalized increase in resistance *R*/*R*_0_ for CuNW films with different transmittances that are exposed to the ambient air is depicted in Figure 4a, over a duration of 25 days. *R*_0_ denotes the resistance value of the CuNW films at ambient conditions, before the different degradation tests are performed. The black squares indicate the data for a CuNW-film with a transmittance of around 85%, whereas the red spheres represent the data for a CuNW film with a transmittance of around 81%. It can be recognized that the film with a high transmittance has almost turned non-conductive after 25 days, whereas the resistance of the film with a low transmittance has increased by only a factor of six. The increase in resistance is attributed to the surface oxidation of the CuNWs [27], which can be recognized by the formation of metal oxide nanoparticles CuNW shell. The formation of metal oxide nanoparticles has also been observed below for the degradation with regard to electrical current and humidity (see Figure A4). This effect is illustrated in Figure 4b that shows the FESEM-image for CuNWs, which were exposed to the ambient atmosphere for more than 25 days. The fact that the film with a lower transmittance and in turn a lower initial resistance shows a reduced change in resistance over time can be understood as follows: The reduced resistance is connected with a higher wire density, which increases the probability that highly conductive paths composed of thick wires and low junction resistances are established. Since the thick wires are more resilient to oxidation, the total resistance of the film is more stable when exposed to the ambient atmosphere or a harsh environment. This behavior is in agreement with the previously published work on random percolating networks from Manning et al., [62], who observed the presence of so called *Winner Takes it All* (WTA) pathways. These WTA pathways are percolating paths with resistances that are significantly lowered compared to other paths and thus carry the major portion of the current. In their study, the lowered resistance was attributed to a lowered junction resistance, whereas for our study, we believe that the lowered resistance stems from the lowered junction resistance and pathways composed of thicker wires.

#### 3.2.2. Elevated Temperatures

**Figure 5:**
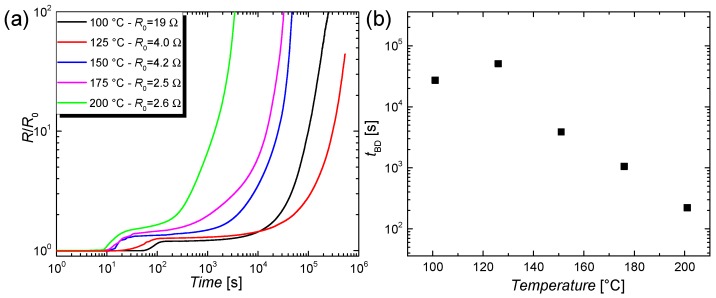
(**a**) Normalized increase in resistance *R*/*R*_0_ over time for different temperatures. The CuNW films were placed on a hot plate in ambient air; (**b**) Breakdown time *t*_BD_ as a function of the annealing temperature.

In this section, the degradation of CuNW films that are subjected to elevated temperatures in a range of 100–200 °C are studied. For this, the films on a glass substrate are placed on a hot plate, under ambient conditions. The films are subsequently heated to various temperatures and their change in resistance with regard to the initial resistance at room temperature is tracked over time, as shown in Figure 5a. To quantify the breakdown behavior, a breakdown time *t*_BD_ is introduced and plotted in Figure 5b as a function of the temperatures. *t*_BD_ is defined as the time when the resistance of the CuNW films has doubled with respect to the initial value. For the higher temperatures, i.e., for 150, 175 and 200 °C, it can be concluded that the increase in resistance goes along with an increase in temperature. However, the change in resistance for the lower temperatures, i.e., for 100 and 125 °C, does not follow the aforementioned trend. This counter-intuitive behavior can be explained by considering the initial resistances of the films. On a qualitative level, it can be argued that a CuNW film with a lowered initial resistance shows a high wire density and thus the effect of chemical degradation induced by oxidation of the nanowire shell seems to be less. Instead, the higher number of junctions lead to a reduced influence of each junction on the complete film behavior on a denser film. To better understand this phenomenon, a model that describes the oxidation of CuNWs and captures the breakdown time, the annealing temperature, and the initial resistance of the films is derived in Section 3.2.6.

#### 3.2.3. Electrical Current

**Figure 6:**
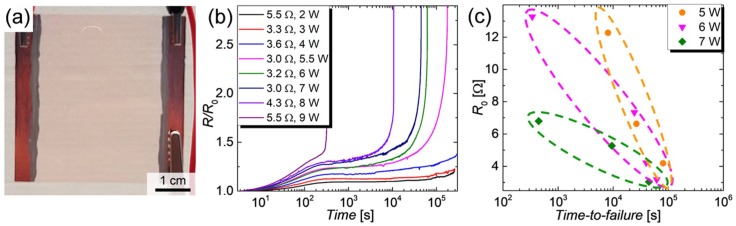
(**a**) Photo of a CuNW film spray-deposited to a glass substrate and electrically contacted on each side by copper tape and conductive silver ink; (**b**) Normalized resistance *R*/*R*_0_ as a function of time for CuNW heaters that show resistances in a range of 3.0 Ω to 5.5 Ω and were subjected to an electrical input power ranging from 2 W to 9 W; (**c**) *R*_0_ as a function of the time-to-failure for CuNW heaters that are subjected to powers of 5 W, 6 W and 7 W, where the ellipses indicate the regions of time-to-failure for same power values.

The degradation of CuNW thin films under current flow will be presented in the following. A photograph for a typical CuNW electrode under test is illustrated in Figure 6a.

**Figure 7:**
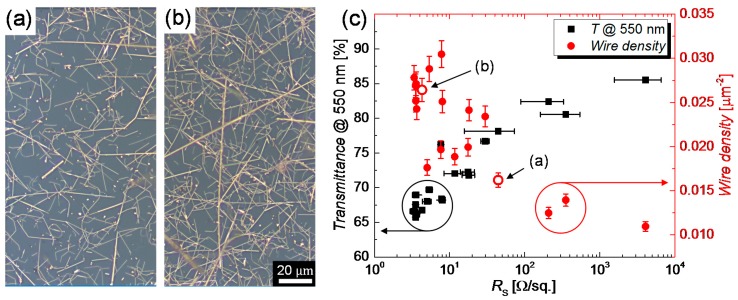
Light microscope images for CuNW films with (**a**) a low and (**b**) a high wire density. (**c**) Transmittance evaluated at a wavelength of 550 nm and wire density as a function of the room temperature resistance. The wire densities corresponding to the images (**a**,**b**) are indicated by red hollow symbols.

For this degradation study, a LabVIEW program was purposely developed to source a constant power in a range of 2 W to 9 W over an effectively heated area of 17.5 cm^2^, via programmable sources, as described in Section 2.6.3. The normalized increase in resistance *R*/*R*_0_ of the CuNW films is depicted in Figure 6b. It can be recognized that up to a power of 4 W the heaters are relatively stable and can be operated for more than 3 days (1 day = 0.86 × 10^5^ s), without device failure. For higher powers, i.e., for 5.5 W, the films show a rapid increase in resistance after a duration of around 8 h, which, quickly results in a device failure. For the change in resistance behavior, three regimes can be identified: (I) a fast increase in resistance in the first 5 min attributed to the temperature-induced change in resistance, (II) a slow and gradual increase in resistance due to the gradual oxidation of the films and finally (III) a fast electrical breakdown of the films that is accompanied by fragmentation for the CuNWs, as discussed below. From the *R*/*R*_0_-plots, a time-to-failure is defined as the time after which the programmable power source applies the maximum programmable voltage of 30 V to maintain the predefined input power. The time-to-failure for CuNW films is shown in Figure 6c. The dotted ellipses indicate the regions of time-to-failures for several initial resistances with the same applied power value. Independent of the applied powers and the initial resistances, the CuNW films undergo a slow and gradual oxidation followed by a rapid breakdown within around 10 s. This behavior is visualized in Figure A5, that depicts the shifted (x-axis) *R*/*R*_0_-time curves for CuNW films at the moment of the breakdown. For the CuNW films it is evident that an increase in power or an increase in the initial resistance leads to a faster degradation and in turn lowers the time-to-failure from almost 1 day for (*P*_1_;*R*_0,1_) = (5 W;4.5 Ω) to 7 min for (*P*_2_;*R*_0,2_) = (7 W;7 Ω). The increase in power leads to an increase in temperature and in turn, enhances the oxidation and the fragmentation of the wires. A higher initial resistance and in turn lower network density also reduces the chemical robustness of the films since the probability of forming robust percolating paths with thick wires decreases with decreasing wire density. In order to verify that the degradation is enhanced for lower network densities and in turn higher initial resistances, as seen in Figure 6c, a wire density has been extracted for all CuNW-films from the microscope images shown in Figure 7 for (a) a low and (b) a high network density.

The transmittances of the films at a wavelength of 550 nm as well as their corresponding wire densities are shown in Figure 7c as a function of the sheet resistance. The data points in Figure 7c that are associated with the low and high network densities illustrated in Figure 7a,b, respectively, are drawn as hollow symbols. In accordance with the expectation, the transmittance shows a decreasing trend with decreasing initial resistance, whereas the wire density increases. The highest electro-optical performance was achieved for a film with a sheet resistance of 7.6 Ω/sq. at a transmittance of 77%. These values yield to a figure of merit (*FoM* = *T*^10^/*R*_S_) of 1000 × *FoM* = 9.6, which follows the commonly accepted definition from Haacke [63]. This FoM compares well to the values reported for metal nanowire-based TEs, which lie in the range of 1000 × *FoM* = 1–30 [14]. Next, two types of current densities that are commonly used in the literature are calculated and allow a comparison with other film heater and degradation studies. The total current *I* sourced to the network, the wire density *n*, and the total area of the heater *A* are used to calculate the current density per nanowire, in agreement with previous works [64]. By division with the cross-section area using the mean diameter of one nanowire *d*, the current density *j* for each individual nanowire can be calculated as follows:

(1) j=I(n·A·π·d24)

**Figure 8:**
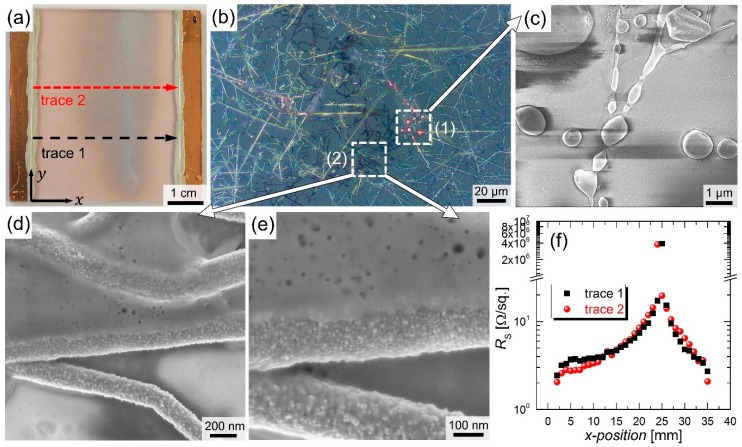
(**a**) Photo for a CuNW heater with a sheet resistance of around 3.2 Ω/sq. after electrical breakdown. The heater was subjected to an electrical input power of 6 W for around 17 h; (**b**) Microscope images for the CuNW film after an electrical breakdown at a position with >MΩ sheet resistance; (**c**–**e**) SEM-images for the CuNW heater shown in (a,b) at a position with >MΩ sheet resistance; (**f**) *R*_S_ across the CuNW heater as a function of the measurement position, as indicated in (**a**).

Depending on the wire density, for a sourced power of around 6 W, a mean current density of around 1.5 to 2.75 MA/m^2^ that is sourced to the wire cross sections in the network is calculated. Another definition for the current density, denoted as *j*_Khaligh_, was introduced by Khaligh et al., as follows [65]:

(2) $$j_{\mathrm{Khaligh}}=\frac{I}{A}$$

where *I* and *A* denote the total current and the effectively heated area, respectively. Following this definition, the current densities calculate to around 10 to 100 mA/cm^2^ for different initial resistance values and sourced powers in the range of 4 W to 9 W. A CuNW-heater that has been subjected to an electrical input power of 6 W until its breakdown, after around a duration of 17 h, is shown in Figure 8a. It should be noted that we considered the current-temperature dependence of the films that heat up due to Joule heating. For this reason, the current values of the heated films and not the ones at room temperature were considered. At an electrical power of 4 W that leads to a temperature of the film of around 100 °C, the resistance increases by a factor of around 1.25, which leads to a reduction in current of 10.5%. For this calculation, we assumed a temperature coefficient of 3.2 mK^−1^ for CuNW networks, which we determined in a previous work [27].

**Figure A3:**
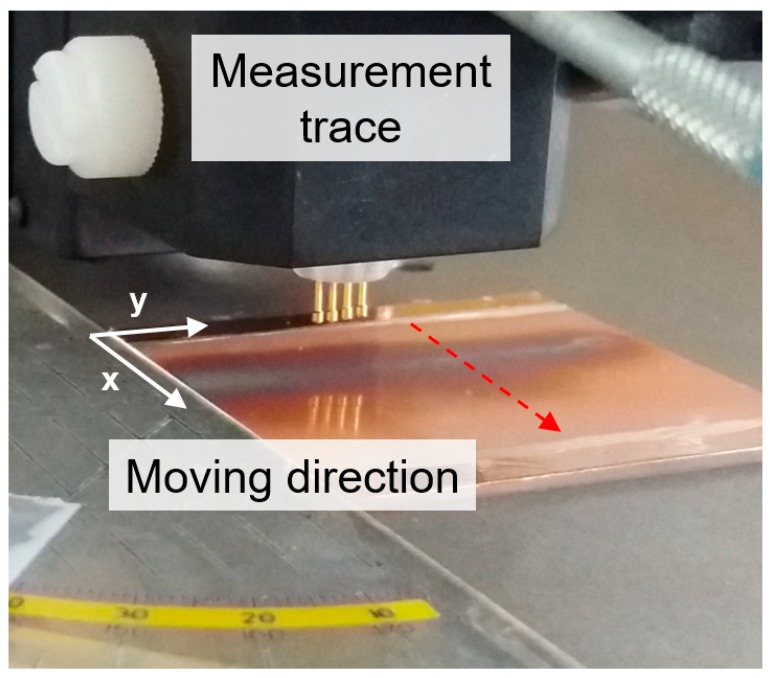
Photo for the four-probe measurement across a CuNW film after electrical breakdown.

The reddish and greyish areas in the degraded film are associated with slightly and heavily oxidized CuNWs, respectively. The greyish appearance of oxidized CuNW films has already been observed in other studies [27]. A microscope image that provides an insight into the greyish section of the film is depicted in Figure 8b. Two main degradation mechanisms are clearly visible and further supported by SEM-images: (c) fragmentation and (d,e) oxidation that goes along with the formation of copper oxide nanoparticles, as observed in Figure 4b for the degradation under ambient air. To correlate the color of the CuNW film with the degree of oxidation, the sheet resistances were measured across the film, along the two measurement traces in x-direction that are indicated in Figure 8a (see Figure A3 for a photo of the four probe needle placement, which was aligned along the y-direction). At the position of the maximum degradation, i.e., where fragmentation can be observed, the film turns non-conductive, whereas in the remaining areas of the heater, the resistance changes from around 3 Ω in the vicinity of the contact leashes to around 20 Ω around the heavily oxidized part. The formation of a crack during the failure of metal nanowire networks under current flow, as shown in Figure 8a, has also been observed in a previous study for the case of AgNWs [66]. In this work, Sannicolo et al. [66] showed that the electrical breakdown takes the form of a global or statistical phenomenon. Further, the authors simulated the formation of a crack during the failure of the network, which propagates parallel to the bias electrodes. These results indicate that a stable operation of the CuNW heaters is possible for more than 3 days at an input power of around 4 W, which corresponds to a current density with respect to the wire cross-section of 1.2 to 2.2 MA/m^2^. The temperature of the CuNW film subjected to a power of 4 W corresponds to a temperature of around 100 °C, in agreement with our previous works for CuNW-based heaters [27,28]. For higher input powers, the fragmentation and oxidation of the CuNWs are readily enhanced.

#### 3.2.4. Moisture

**Figure 9:**
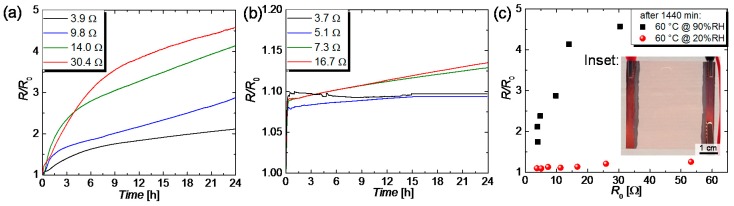
*R*/*R*_0_ plotted as a function of time for CuNW heaters with different *R*_0_ that were subjected to different moisture levels of (**a**) 90%RH and (**b**) 20%RH, at a temperature of 60 °C and for a duration of 24 h. (**c**) *R*/*R*_0_ as a function of *R*_0_ for the two moisture levels. The inset shows a photo for a CuNW film.

For the degradation study of sprayed CuNW films with regard to elevated moisture levels, the films are placed in a climatic chamber, at a constant temperature of 60 °C, and the relative moisture is varied. The normalized increase in resistance *R*/*R*_0_ is depicted in Figure 9 for two different moisture levels of (a) 90%RH and (b) 20%RH.

**Figure A4:**
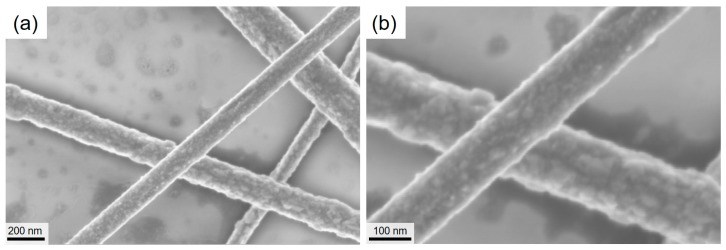
SEM-images for a CuNW film subjected to a relative humidity and temperature of 90%RH and 60 °C, for a duration of 24 h, under (**a**) a low and (**b**) a high magnification.

**Figure A5:**
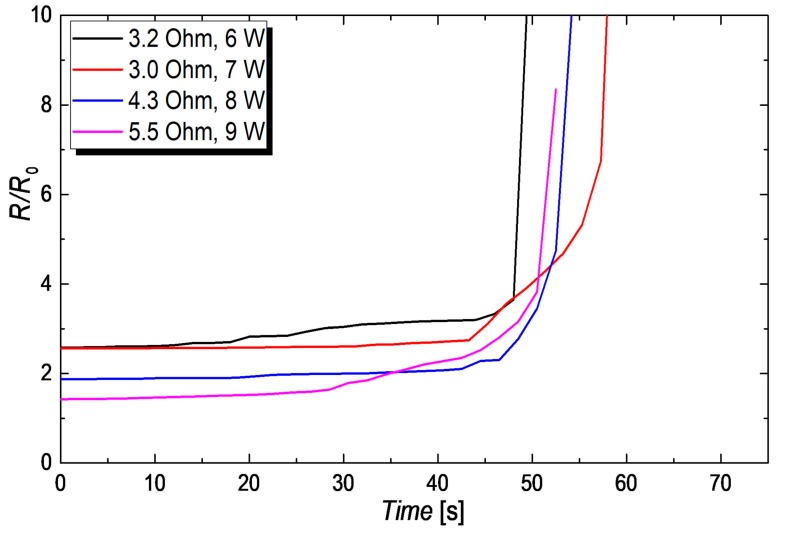
Normalized increase in resistance *R*/*R*_0_ over time for CuNW films with a different initial resistance that were subjected to different electrical input powers. The x-axes for all films have been shifted to allow comparing the breakdown regions of each device.

After a duration of 24 h, the resistances of the CuNW films subjected to a temperature of 60 °C and a relative moisture of 90%RH increased up to around a factor of 2 and 4.5, respectively, for the films with the lowest and the highest initial resistance. This result is in accordance with the expectation that films with a lowered initial resistance are more robust to degradation, which reflects in a reduced increase in resistance. Figure 9b shows *R*/*R*_0_ as a function of the time for CuNW films subjected to a temperature of 60 °C and a humidity of 20%RH. In the beginning, the resistance of the CuNW rises fast, attributed to the resistance-temperature dependence of copper. After the climatic chamber has reached its target temperature of 60 °C, the films show a gradual increase in resistance of around 1 to 4%, for a total duration of 24 h. Similar to the behavior of the high relative moisture of 90%RH, the films with a higher initial resistance are more prone to degradation. For the films subjected to the high humidity value, the formation of metal nanoparticles could be seen, as shown in Figure A4, and observed for the case of the exposure to the ambient air (see Figure 4b) and electrical current (see Figure 8d,e). The aforementioned results are summarized in Figure 9c that shows *R*/*R*_0_ as a function of the initial resistance *R*_0_ for the two different relative moistures. It can be concluded that the degradation is greatly enhanced for the high relative moisture of 90%RH, which leads to an increase in resistance that is around a factor of 100 larger than for the low relative moisture of 20%RH.

#### 3.2.5. UV Light

**Figure 10:**
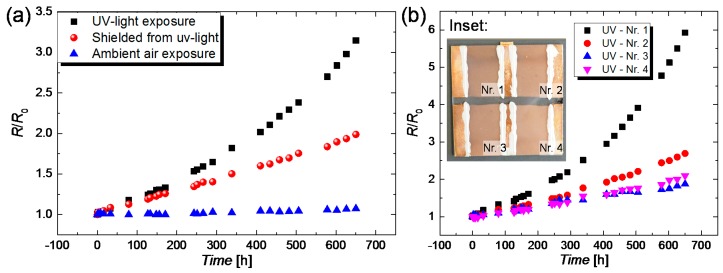
(**a**) Normalized increase in resistance *R*/*R*_0_ over time for CuNW films with a transmittance of 70% that were (i) subjected to ultraviolet-visible (UV-vis) light, (ii) shielded from UV-vis light and (iii) exposed to the ambient air. Each symbol represents a mean resistance that was determined by averaging over four different samples; (**b**) *R*/*R*_0_ over time for four different CuNW films that are subjected to prolonged UV-vis exposure. The inset depicts the four CuNW samples that were subjected to UV-vis exposure along with a labeling that is in accordance with normalized resistance curves.

Since CuNWs are also intended to be integrated to touch panels or into aircraft and car windows, their degradation during the exposure to ultraviolet-visible (UV-vis) light has to be investigated. To study the UV-vis-light-induced degradation, CuNW films with comparable initial resistances and a transmittance of around 70% were placed in a UV-vis box that is described in Section 2.6.5, under (i) direct light exposure and (ii) shielded from the light by a thin aluminum plate. The normalized resistances over time for CuNW films subjected to both aforementioned stresses are depicted in Figure 10a. For a better separation of the degradation mechanism, the resistances for samples that are subjected to the ambient air are also characterized and the results are plotted in Figure 10a. It should be noted that, for each stress, the resistances of four different CuNW films that are exemplarily illustrated in the inset in Figure 10b for the case of exposure to UV-vis-light, were considered. From Figure 10a it can be seen that the increase in resistance is more pronounced for the films placed in the UV-vis Box than for the ones placed in the ambient air. It should be noted that the CuNW films subjected to the ambient air, as shown in Figure 10a, are more robust than the ones characterized in Figure 4a. The difference in the degree of degradation can be attributed to the different transmittance values of the films, which were significantly lower for the UV-treated samples, i.e., around 70% compared to the 80% and 85% for the films shown in Figure 4a. The increased degradation for the CuNW films shielded from the light stems from an increased temperature in the box of around 60 °C, as measured using a Pt100 thermoresistor. The increase in resistance of the CuNW films subjected to UV-vis light is larger than the increase for the shielded samples, which indicates that UV-vis light, in fact, induces a degradation for CuNWs, as previously reported [61]. In the literature, papers that report on the UV-induced degradation of polymers [67,68] and silver nanoparticle [69] can be found. However, so far, no study gives an explanation for this effect, for the case of CuNWs.

We strongly believe that the degradation arises from free reactive oxygen species (ROS), generated by longwave UV light (UVA) in the range of 320–400 nm, as reported by Herrling et al., [70] and other groups [71]. A higher ROS concentration is accompanied by an increased oxidation of the metal and subsequently a faster degradation of the CuNW films. An additional degradation process can be induced by an increase in temperature. This increase in temperature can be attributed to the generation of surface and bulk plasmon polaritons that are predominantly formed by absorption of light in the UV-vis spectrum, below a wavelength of 500 nm [72,73].

#### 3.2.6. Discussion and Modeling of the Temperature-Induced Oxidation

**Figure 11:**
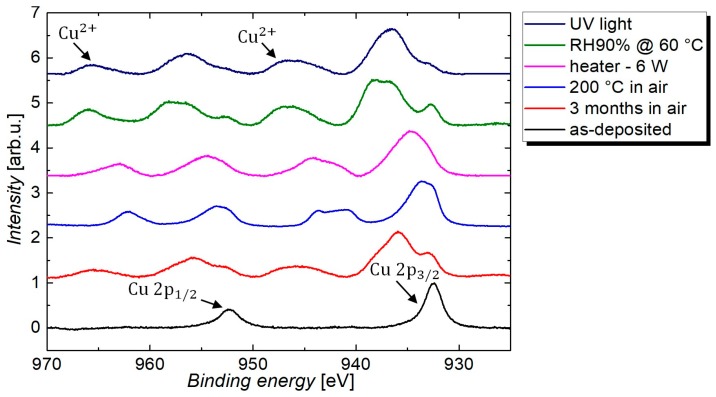
X-ray photoelectron spectroscopy (XPS) spectra for CuNW film that were subjected to different environmental stresses, under ambient air, i.e., (1) as-deposited, (2) exposure to ambient air for 3 months, (3) subjected to a temperature of 200 °C for a duration of 30 min, (4) heated at a power of 6 W until breakdown, (5) subjected to a relative humidity of 90%RH, at a temperature of 60 °C, and (6) UV-light exposure.

The degradation tests showed that the increase in resistance under ambient air is greatly increased for higher temperatures. As reported in previous works, the degradation of CuNWs in ambient air stems from the oxidation of the nanowire shell to copper oxide [49]. However, to date, there is no study for CuNWs that discusses the formation of two oxide shells with a different chemical composition on the CuNWs and presents a model for the time- and temperature-dependence of the oxide thickness, which will be discussed below. Via X-ray photoelectron spectroscopy (XPS), the oxidation of the CuNW films was studied in more detail, as shown in Figure 11, that depicts the high-resolution Cu 2p core-level spectra for CuNW films subjected to all the environmental stresses that were discussed in this paper. For the as-deposited CuNW films, no oxygen-related contribution could be identified from the Cu 2p peak, whereas for all other samples, a clear appearance of copper shake-up peaks that are centered around a binding energy of 942 eV and 962 eV can be recognized. This appearance of shake-up peaks along with a shift of the Cu 2p_1/2_ and Cu 2p_3/2_ peak to higher binding energies is a proof for the predominant formation of divalent copper species, i.e., CuO [74,75,76]. Further, a larger shift of the peaks to higher binding energies indicates a higher degree of oxidation.

**Figure 12:**
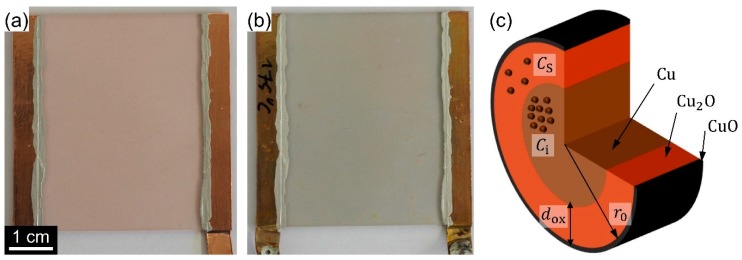
Photos for (**a**) an as-deposited CuNW film and (**b**) a CuNW film subjected to a temperature of 175 °C for a duration of 1 h. (**c**) Schematic of an oxidized CuNW along with the parameters that are used in the text to describe the oxidation mechanism.

This oxidation of the CuNW film also becomes evident from its greyish appearance, as shown in Figure 12 for the photos of (a) an as-deposited CuNW film and (b) a CuNW film subjected to a temperature of 175 °C for a duration of 1 h. However, there is no study that reports on a model or a quantitative description for the oxidation mechanism of CuNWs. As sketched in Figure 12c, the corroded CuNW shell is known to consist of a thick inner shell of cuprous oxide (Cu_2_O) that, on the outer face, reacts further to cupric oxide (CuO) [77,78]. The formation of CuO on the outermost shell of the CuNWs, which is in agreement with the literature, is also in accordance with the XPS spectra shown in Figure 11 that indicate that mostly CuO is present. 

The chemical reactions for the formation of Cu_2_O and CuO are given in Equations (3) and (4).

(3) $$4\mathrm{Cu}+O_{2}\to 2{\mathrm{Cu}}_{2}O$$

(4) $$2{\mathrm{Cu}}_{2}O+O_{2}\to 4\mathrm{Cu}O$$

For the oxidation of thin copper films, which should have chemical properties similar to the ones of CuNWs, it has been discovered that (i) the outer CuO layer with a thickness in the range of 10–50 nm is much thinner than the Cu_2_O layer; (ii) the growth of the copper oxide shell is mainly due to the growth and expansion of the Cu_2_O layer, and (iii) the growth mechanism is a diffusion-controlled process that will be discussed in more detail in the following [79,80]. In the case of copper, the out-diffusion of copper atoms and their subsequent reaction with oxygen dictates the oxidation process [81,82]. This mechanism is in contrast to the well-studied oxidation of silicon [83], where the diffusion of oxygen occurs in the ambient air through the silicon. The driving force for the oxidation of copper is grain-boundary-diffusion, which in turn depends on the size of the individual grains [38]. In agreement with the literature, the temperature-dependent grain-boundary-diffusion constant is described using the Arrhenius equation [84]:

*D* = *D*_0_exp(−*E*/*k*_B_*T*) (5)

where *D*_0_ denotes the pre-exponential factor of grain-boundary diffusion, *E* the activation energy, *k*_B_ the Boltzmann constant and *T* the temperature, respectively. Next, an expression for the time-dependent oxide thickness *d*_ox_(t) is derived. As a starting point, Fick’s law is employed to describe the diffusion *F*_d_ of copper atoms, which is dictated by the concentration gradient of the copper atoms *C*_i_ in the non-oxidized and *C*_s_ the oxidized region of the CuNW, as sketched in Figure 12c:

(6) $$F_{d}=D/d_{\mathrm{ox}}(t)\cdot$$

**Figure 13:**
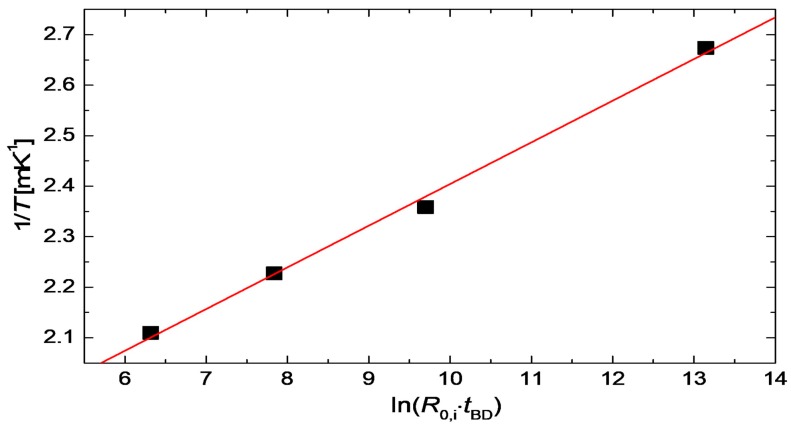
Temperature-breakdown time dependence for the data from Figure 5b. The solid line represents a linear fit to the experimental data, in agreement with Equation (10).

After some math and physical assumptions that are described in more detail in Appendix C, a parabolic rate law is derived for the time-dependent oxide thickness, as follows:

(7) $$d\mathrm{ox}\left(t\right)=\sqrt{B\cdot t}$$

where *B* denotes a reaction-specific constant, in accordance with the Deal-Grove model [83]. After deriving the parabolic law for the oxide thickness in Equation (7) and having in mind that the diffusion of copper atoms is described by the Arrhenius law in Equation (5), a relation between the breakdown-time *t*_BD_, as shown in Figure 5b, and the annealing temperature *T* can be derived. For simplicity, it is assumed that the resistance of the different CuNW films *R*_0,i_ can be modeled by the resistance of a metallic cylinder with the formula:

(8) $$R_{0,i}=\rho \cdot \frac{l}{A}$$

where *ρ* denotes the bulk resistivity of copper and *l* and *A* denotes the length and the cross-section of the conductor. It has to be noted that Equation (8) represents a drastic simplification for the modeling of the film resistance and is only employed for a qualitative description of the oxidation process. During the oxidation, the CuNW shell is consumed to copper oxide and the cross section that can effectively contribute to the electrical percolation is reduced. Hence, a time-dependent resistance *R*(t) that depends on the time-dependent oxide thickness *d*_ox_(t) is given by:

(9) $$R(t)=\rho \cdot l/\pi {\left(r_{0}-d_{\mathrm{ox}}\left(t\right)\right)}^{2}$$

where *r*_0_ denotes the initial radius of the pristine nanowire, as sketched in Figure 12c. After plugging Equation (7) in Equation (9) and substituting the reaction specific constant *B* with the Arrhenius Equation (5) (see Appendix C for more details), an analytical dependence can be given for the temperature-breakdown time dependence, as follows:

(10) $$\frac{1}{T}=a\cdot \ln\left(\sqrt{b\cdot R_{0,i}\cdot t_{\mathrm{BD}}}\right)+c$$

where *a*, *b* and *c* denote constants. To verify the validity of this equation for the degradation of CuNWs, the annealing temperatures and breakdown times from Figure 5b were plotted in Figure 13. The graph shows a clear linear dependence, which proves that the derived temperature dependence for the breakdown time agrees well with our model.

The presented model can help to predict the breakdown of CuNW-based devices if the process temperature and the initial film resistance are known.

### 3.3. Encapsulation of Copper Nanowires

**Figure 14:**
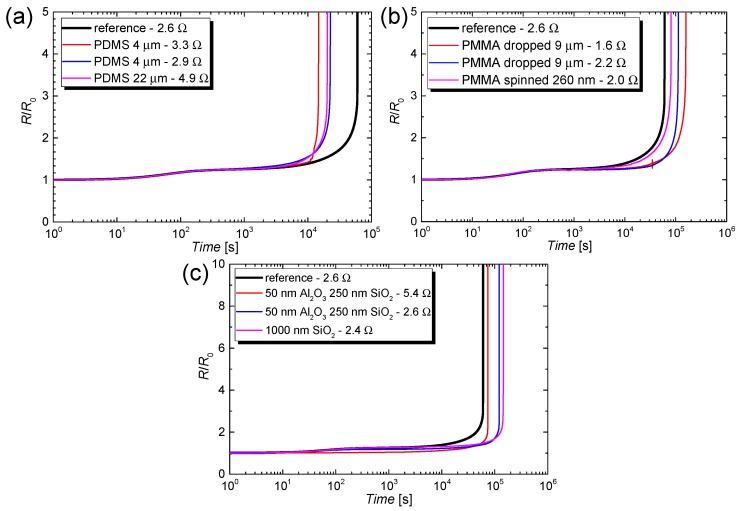
Normalized increase in resistance *R*/*R*_0_ as a function of the time for CuNW films coated with (**a**) PDMS, (**b**) PMMA and (**c**) SiO_2_ and Al_2_O_3_. The CuNW films were heated over an effective area of 3.5 × 5 cm^2^ by applying an electrical input power of 6 W.

Despite the high relevance of this aspect, there are only a very few studies that report on the encapsulation of CuNW films [30,49,50], as highlighted in the introduction section. In this work, we test various coating materials such as PDMS, PMMA and electron beam evaporated oxides as coating materials. The aim of this study is to increase the lifetime of CuNW-based devices under current flow or at elevated temperatures and to compare the effectiveness of a low-cost polymer-based coating with an expensive ebeam evaporated oxide coating. The normalized increase *R*/*R*_0_ as a function of the time for CuNW films subjected to an electrical input power of 6 W is depicted in Figure 14 for the coating materials (a) PDMS, (b) PMMA, and (c) SiO_2_ and Al_2_O_3_.

The resistance transient for an uncoated CuNW film is plotted in the graphs (a–c) and is denoted as the reference. So far, PDMS has been used as a substrate material for CuNWs [85] but little is known about its performance as an encapsulation material. In Figure 14 it can be seen that the PDMS coating leads to a reduction in lifetime from around 17 h for the reference film to around 4–6 h for the films coated with a thickness of either 4 or 22 µm. On the one hand, this behavior can be attributed to the high permeation rate of PDMS for gases in the ambient air [86]. On the other hand, since the reduction in lifetime is drastic, the degradation could also be induced by a chemical interaction between the PDMS and the CuNW film. From the *R*/*R*_0_ response of CuNW films coated with PMMA, it can be concluded that a thin film with a thickness of 200 nm, applied by spin coating, has no significant effect as an encapsulation material. For thicker films with a thickness of around 9 µm, the lifetimes of the CuNW networks were 31 h and 44 h, respectively, which corresponds to an increase by 82% and 160%, respectively, compared to the lifetime of the reference film. This result is in agreement with the work of other groups who successfully tested PMMA as a coating material for CuNW films [49,61]. As the last coating materials, electron beam evaporated SiO_2_ and Al_2_O_3_ were, to the best of our knowledge, for the first time tested in combination with CuNWs. From Figure 14c it can be seen that the oxide coatings lead to a lifetime of 21 h, 34 h (both encapsulations are composed of 50 nm Al_2_O_3_ & 250 nm SiO_2_) and 40 h (1000 nm SiO_2_), respectively, which corresponds to an increase in lifetime of 24%, 100% and 135%, respectively, with regard to the reference sample. A combination of SiO_2_ and Al_2_O_3_ was tested since stacks composed of different oxide layers were reported to show a lowered gas permeability. This effect is in accordance with the work from Dameron et al., [87] who found that the evaporation of SiO_2_ onto Al_2_O_3_ can heal out defects in the Al_2_O_3_ film that could otherwise serve as diffusion sides for the permeation of ambient gases.

## 4. Conclusions

In summary, we investigated the up-scaling potential of an aqueous synthesis of CuNWs, and studied the degradation and the encapsulation of sprayed CuNW films. For the synthesis, we have observed that the precursor-to-solvent weight ratio should be kept below 1:100 to allow a stable micelle formation of the copper-oleylamine complex during growth and to improve the dispersibility of the nanowires. To the best of our knowledge, our work is the first report on the effect of the precursor-to-solvent ratio, which is an important factor for the upscaling potential of the presented synthesis. For the commercialization of CuNWs, the upscaling potential is a key factor, since there are various different applications requiring TEs such as heaters, touch panels, solar cells or OLEDs. To substitute ITO in all mentioned devices, large amounts of CuNWs are needed. Further, we rigorously studied the degradation mechanism for CuNWs, which will serve as a reference in future to explain and predict the failure of CuNW-based devices in follow-up studies. For the CuNW film degradation, we observed that chemical degradation via oxidation is the main degradation mechanism, as observed in other studies. We believe that the UV-driven degradation of CuNWs, that has not yet been understood in the literature stems from the UV-induced generation of ozone that leads to rapid oxidation of the CuNWs. In addition to these degradation studies, a purposely-developed model was presented to correlate the breakdown time of the CuNW film with the temperature and the initial resistance of the films. The encapsulation of the CuNW films using polymers such as PDMS proved to be difficult. In contrast to the expectation, the PDMS coating lowered the lifetime of the films, whereas a positive trend could be observed for PMMA, in agreement with the literature, as well as for electron beam evaporated oxide coatings that have been tested for the first time. We found that the maximum improvement in the lifetime is comparable for PMMA and ebeam evaporated oxides. Therefore, for economic reasons, PMMA represents a promising coating material since it can be applied at a large scale, under ambient conditions, and the material costs are also low. To further increase the lifetime of CuNW-based devices, we propose to use a combination of two techniques that have already been tested with success: The laser-induced nanowelding of CuNW junctions, followed by the sealing of the film with a PMMA encapsulation.

## Appendix C. Oxidation Model for CuNWs

The physical assumption applied during the derivation of the time dependence of the oxide thickness is a variant of the so-called Deal-Grove Model. The underlying principle of the model is the equality of the diffusion flux of copper atoms and the reaction rate of the oxidation process. The diffusion flux *F*_d_ is described by Equation (A1), as follows:

(A1) $$F_{d}=\frac{D}{d_{\mathrm{ox}}\left(t\right)}\cdot \left(C_{s}-C_{i}\right)$$

where *D* denotes the diffusion constant, *d*_ox_(*t*) the time-dependent oxide thickness and *C*_s_ and *C*_i_ the concentration of copper atoms in the copper oxide shell and in the pristine copper, respectively, in agreement with the schematic for the oxidation of a single nanowire in Figure 12. The reaction-rate *F*_r_ is defined by Equation (2).

(A2) $$F_{r}=M\cdot \frac{d}{dt}d_{\mathrm{ox}}\left(t\right)=k_{s}\cdot C_{i}$$

where *M* is the oxygen density in the oxide layer and *k*_s_ is the reaction-rate coefficient. Equations (A1) and (A2) together yield to the differential expression for the oxide thickness of:

(A3) $$\frac{d}{dt}d_{\mathrm{ox}}\left(t\right)=k_{s}\cdot \frac{C_{i}}{M}=k_{s}\cdot \frac{C_{s}}{M\cdot \left(1+\frac{k_{s}}{D}\cdot d_{\mathrm{ox}}\left(t\right)\right)}$$

This differential equation can be solved under the assumption that *d*_ox_(*t*) is much larger than *D*/*k*_s_, which yields to the parabolic rate law that is also derived in the main text.

(A4) $$d_{\mathrm{ox}}\left(t\right)=\sqrt{B\cdot t};B=2\cdot \frac{D\cdot C_{s}}{M}$$

In the following, the equation that relates the annealing temperature with the initial resistance and the breakdown time, i.e., Equation (10) in the main text, is derived in more detail. In Equation (9), the nanowire radius *r*_0_ can be substituted by:

(A5) $$r_{0}=\sqrt{\pi \cdot \frac{R_{0}}{\rho \cdot L}}$$

where *R*_o_ and *L* denote by the effective length and resistance of the nanowire network and *ρ* the resistivity of copper, respectively. For the time-dependent oxide thickness *d*_ox_(*t*) in Equation (9), the parabolic rate law in Equation (A4) is utilized, which gives the following equation:

(A6) $$\frac{R_{t}}{R_{0}}=\frac{1}{{\left(1-\sqrt{\pi \frac{R_{0}}{\rho L}Bt}\right)}^{2}}$$

As described in Equation (A4) in the supporting information and Equation (5) in the main text, the following expression is derived for *B*:

(A7) $$B=D\cdot e^{\frac{-E}{kT}}\to \sqrt{\frac{\rho }{R_{0}t_{bd}}}e^{\frac{-E}{2kT}}$$

when the expression for *B* provided in Equation (A7) is plugged into Equation (A6), the equation for the initial resistance-breakdown time dependence of the annealing temperature in Equation (10) in the main text can be derived.

## References

[1] Li J., Hu L., Wang L., Zhou Y., Grüner G., Marks T.J. (2006). Organic light-emitting diodes having carbon nanotube anodes. Nano Lett..

[2] Vosgueritchian M., Lipomi D.J., Bao Z. (2012). Highly conductive and transparent PEDOT:PSS films with a fluorosurfactant for stretchable and flexible transparent electrodes. Adv. Funct. Mater..

[3] Li X., Zhu Y., Cai W., Borysiak M., Han B., Chen D., Piner R.D., Colombo L., Ruoff R.S. (2009). Transfer of large-area graphene films for high-performance transparent conductive electrodes. Nano Lett..

[4] Yin Z., Sun S., Salim T., Wu S., Huang X., He Q., Lam Y.M., Zhang H. (2010). Organic photovoltaic devices using highly flexible reduced graphene oxide films as transparent electrodes. ACS Nano.

[5] Meng L., dos Santos M. (1998). Properties of indium tin oxide films prepared by rf reactive magnetron sputtering at different substrate temperature. Thin Solid Films.

[6] Sun Y., Gates B., Mayers B., Xia Y. (2002). Crystalline silver nanowires by soft solution processing. Nano Lett..

[7] Rathmell A.R., Bergin S.M., Hua Y.L., Li Z.Y., Wiley B.J. (2010). The growth mechanism of copper nanowires and their properties in flexible, transparent conducting films. Adv. Mater..

[8] Dou L., Cui F., Yu Y., Khanarian G., Eaton S.W., Yang Q., Resasco J., Schildknecht C., Schierle-Arndt K., Yang P. (2016). Solution-processed copper/reduced-graphene-oxide core/shell nanowire transparent conductors. ACS Nano.

[9] Tokuno T., Nogi M., Jiu J., Suganuma K. (2012). Hybrid transparent electrodes of silver nanowires and carbon nanotubes: A low-temperature solution process. Nanoscale Res. Lett..

[10] Stapleton A.J., Afre R.A., Ellis A.V., Shapter J.G., Andersson G.G., Quinton J.S., Lewis D.A. (2013). Highly conductive interwoven carbon nanotube and silver nanowire transparent electrodes. Sci. Technol. Adv. Mater..

[11] Chen X., Guo W., Xie L., Wei C., Zhuang J., Su W., Cui Z. (2017). Embedded Ag/Ni metal-mesh with low surface roughness as transparent conductive electrode for optoelectronic applications. ACS Appl. Mater. Interfaces.

[12] Kang M.G., Joon Park H., Hyun Ahn S., Jay Guo L. (2010). Transparent Cu nanowire mesh electrode on flexible substrates fabricated by transfer printing and its application in organic solar cells. Sol. Energy Mater. Sol. Cells.

[13] Khan A., Lee S., Jang T., Xiong Z., Zhang C., Tang J., Guo L.J., Wen-Di L. (2016). High-performance flexible transparent electrode with an embedded metal mesh fabricated by cost-effective solution process. Small.

[14] Langley D., Giusti G., Mayousse C., Celle C., Bellet D., Simonato J.-P.P. (2013). Flexible transparent conductive materials based on silver nanowire networks: A review. Nanotechnology.

[15] Li S., Chen Y., Huang L., Pan D. (2014). Large-scale synthesis of well-dispersed copper nanowires in an electric pressure cooker and their application in transparent and conductive networks. Inorg. Chem..

[16] Lee J.H., Lee P., Lee D., Lee S.S., Ko S.H. (2012). Large-scale synthesis and characterization of very long silver nanowires via successive multistep growth. Cryst. Growth Des..

[17] Scardaci V., Coull R., Lyons P.E., Rickard D., Coleman J.N. (2011). Spray deposition of highly transparent, low-resistance networks of silver nanowires over large areas. Small.

[18] Ye S., Rathmell A.R., Chen Z., Stewart I.E., Wiley B.J. (2014). Metal nanowire networks: The next generation of transparent conductors. Adv. Mater..

[19] Bobinger M., Dergianlis V., Becherer M., Lugli P. (2018). Comprehensive synthesis study of well-dispersed and solution-processed metal nanowires for transparent heaters. J. Nanomater..

[20] Kwon J., Suh Y.D., Lee J., Lee P., Han S., Hong S., Yeo J., Lee H., Ko S.H. (2018). Recent progress in silver nanowire based flexible/wearable optoelectronics. J. Mater. Chem. C.

[21] Jung J., Lee H., Ha I., Cho H., Kim K.K., Kwon J., Won P., Hong S., Ko S.H. (2017). Highly stretchable and transparent electromagnetic interference shielding film based on silver nanowire percolation network for wearable electronics applications. ACS Appl. Mater. Interfaces.

[22] Jeong S., Cho H., Han S., Won P., Lee H., Hong S., Yeo J., Kwon J., Ko S.H. (2017). High efficiency, transparent, reusable, and active PM2.5 filters by hierarchical Ag nanowire percolation network. Nano Lett..

[23] Lee J., An K., Won P., Ka Y., Hwang H., Moon H., Kwon Y., Hong S., Kim C., Lee C. (2017). A dual-scale metal nanowire network transparent conductor for highly efficient and flexible organic light emitting diodes. Nanoscale.

[24] Oh J.Y., Lee D., Jun G.H., Ryu H.J., Hong S.H. (2017). High conductivity and stretchability of 3D welded silver nanowire filled graphene aerogel hybrid nanocomposites. J. Mater. Chem. C.

[25] Lee H., Hong S., Lee J., Suh Y.D., Kwon J., Moon H., Kim H., Yeo J., Ko S.H. (2016). Highly stretchable and transparent supercapacitor by ag-au core-shell nanowire network with high electrochemical stability. ACS Appl. Mater. Interfaces.

[26] Hong S., Lee H., Lee J., Kwon J., Han S., Suh Y.D., Cho H., Shin J., Yeo J., Ko S.H. (2015). Highly stretchable and transparent metal nanowire heater for wearable electronics applications. Adv. Mater..

[27] Bobinger M., Mock J., La Torraca P., Becherer M., Lugli P., Larcher L. (2017). Tailoring the aqueous synthesis and deposition of copper nanowires for transparent electrodes and heaters. Adv. Mater. Interfaces.

[28] Bobinger M.R.R., La Torraca P., Mock J., Becherer M., Cattani L., Angeli D., Larcher L., Lugli P. (2018). Solution-processing of copper nanowires for transparent heaters and thermo-acoustic loudspeakers. IEEE Trans. Nanotechnol..

[29] Bobinger M., Mock J., Becherer M., Torraca P.L., Angeli D., Larcher L., Lugli P. (2017). Characterization and modelling of transparent heaters based on solution-processed copper nanowires. Proceedings of the 2017 IEEE 17th International Conference on Nanotechnology, NANO 2017.

[30] Chen Z., Ye S., Stewart I.E., Wiley B.J. (2014). Copper nanowire networks with transparent oxide shells that prevent oxidation without reducing transmittance. ACS Nano.

[31] Bobinger M., Angeli D., Colasanti S., La Torraca P., Larcher L., Lugli P. (2017). Infrared, transient thermal, and electrical properties of silver nanowire thin films for transparent heaters and energy-efficient coatings. Phys. Status Solidi.

[32] Xiao L., Chen Z., Feng C., Liu L., Bai Z.Q., Wang Y., Qian L., Zhang Y., Li Q., Jiang K. (2008). Flexible, stretchable, transparent carbon nanotube thin film loudspeakers. Nano Lett..

[33] La Torraca P., Bobinger M., Pavan P., Becherer M., Zhao S., Koebel M., Cattani L., Lugli P., Larcher L. (2018). High efficiency thermoacoustic loudspeaker made with a silica aerogel substrate. Adv. Mater. Technol..

[34] Yu Z., Li L., Zhang Q., Hu W., Pei Q. (2011). Silver nanowire-polymer composite electrodes for efficient polymer solar cells. Adv. Mater..

[35] Zaiba S., Kouriba T., Ziane O., Stéphan O., Bosson J., Vitrant G., Baldeck P.L. (2012). Metallic nanowires can lead to wavelength-scale microlenses and microlens arrays. Opt. Express.

[36] Kirsch N.J., Vacirca N.A., Plowman E.E., Kurzweg T.P., Fontecchio A.K., Dandekar K.R. Optically transparent conductive polymer rfid meandering dipole antenna. Proceedings of the 2009 IEEE International Conference on RFID.

[37] Falco A., Cinà L., Scarpa G., Lugli P., Abdellah A. (2014). Fully-sprayed and flexible organic photodiodes with transparent carbon nanotube electrodes. ACS Appl. Mater. Interfaces.

[38] Lee J., Lee P., Lee H., Lee D., Lee S.S., Ko S.H. (2012). Very long Ag nanowire synthesis and its application in a highly transparent, conductive and flexible metal electrode touch panel. Nanoscale.

[39] Lee T.-W., Lee S.-E., Jeong Y.G. (2016). Highly effective electromagnetic interference shielding materials based on silver nanowire/cellulose papers. ACS Appl. Mater. Interfaces.

[40] Park T., Kim B., Kim Y., Kim E. (2014). Highly conductive PEDOT electrodes for harvesting dynamic energy through piezoelectric conversion. J. Mater. Chem. A.

[41] Park T., Na J., Kim B., Kim Y., Shin H., Kim E. (2015). Photothermally activated pyroelectric polymer films for harvesting of solar heat with a hybrid energy cell structure. ACS Nano.

[42] Chang Y., Lye M.L., Zeng H.C. (2005). Large-scale synthesis of high-quality ultralong copper nanowires. Langmuir.

[43] Zhao S., Han F., Li J., Meng X., Huang W., Cao D., Zhang G., Sun R., Wong C.P. (2018). Advancements in copper nanowires: synthesis, purification, assemblies, surface modification, and applications. Small.

[44] Hwang C., An J., Choi B.D., Kim K., Jung S.-W., Baeg K.-J., Kim M.-G., Ok K.M., Hong J. (2016). Controlled aqueous synthesis of ultra-long copper nanowires for stretchable transparent conducting electrode. J. Mater. Chem. C.

[45] Yang H.-J., He S.-Y., Tuan H.-Y. (2014). Self-seeded growth of five-fold twinned copper nanowires: Mechanistic study, characterization, and SERS applications. Langmuir.

[46] Jin M., He G., Zhang H., Zeng J., Xie Z., Xia Y. (2011). Shape-controlled synthesis of copper nanocrystals in an aqueous solution with glucose as a reducing agent and hexadecylamine as a capping agent. Angew. Chem. Int. Ed..

[47] Ye S., Stewart I.E., Chen Z., Li B., Rathmell A.R., Wiley B.J. (2016). How copper nanowires grow and how to control their properties. Acc. Chem. Res..

[48] Ye S., Rathmell A.R., Ha Y.-C., Wilson A.R., Wiley B.J., Ye S., Rathmell A.R., Wilson A.R., Wiley B.J., Ha Y. (2014). The role of cuprous oxide seeds in the one-pot and seeded syntheses of copper nanowires. Small.

[49] Zhai H., Wang R., Wang X., Cheng Y., Shi L., Sun J. (2016). Transparent heaters based on highly stable Cu nanowire. Nano Res..

[50] Chen J., Zhou W., Chen J., Fan Y., Zhang Z., Huang Z., Feng X., Mi B., Ma Y., Huang W. (2015). Solution-processed copper nanowire flexible transparent electrodes with PEDOT:PSS as binder, protector and oxide-layer scavenger for polymer solar cells. Nano Res..

[51] Kim D., Kwon J., Jung J., Kim K., Lee H. (2018). A Transparent and flexible capacitive-force touch pad from high-aspect-ratio copper nanowires with enhanced oxidation resistance for applications in wearable electronics. Small Methods.

[52] Han S., Hong S., Ham J., Yeo J., Lee J., Kang B., Lee P., Kwon J., Lee S.S., Yang M.Y. (2014). Fast plasmonic laser nanowelding for a Cu-nanowire percolation network for flexible transparent conductors and stretchable electronics. Adv. Mater..

[53] Han S., Hong S., Yeo J., Kim D., Kang B., Yang M.Y., Ko S.H. (2015). Nanorecycling: Monolithic integration of copper and copper oxide nanowire network electrode through selective reversible photothermochemical reduction. Adv. Mater..

[54] Park J.H., Han S., Kim D., You B.K., Joe D.J., Hong S., Seo J., Kwon J., Jeong C.K., Park H.J. (2017). Plasmonic-tuned flash Cu nanowelding with ultrafast photochemical-reducing and interlocking on flexible plastics. Adv. Funct. Mater..

[55] Herrera-Gomez A., Bravo-Sanchez M., Ceballos-Sanchez O., Vazquez-Lepe M.O. (2014). Practical methods for background subtraction in photoemission spectra. Surf. Interface Anal..

[56] Lee E.-J., Kim Y.-H., Hwang D.K., Choi W.K., Kim J.-Y. (2016). Synthesis and optoelectronic characteristics of 20 nm diameter silver nanowires for highly transparent electrode films. RSC Adv..

[57] Araki T., Jiu J., Nogi M., Koga H., Nagao S., Sugahara T., Suganuma K. (2014). Low haze transparent electrodes and highly conducting air dried films with ultra-long silver nanowires synthesized by one-step polyol method. Nano Res..

[58] Hotaling N.A., Bharti K., Kriel H., Simon C.G., Simon C.G. (2015). DiameterJ: A validated open source nanofiber diameter measurement tool. HHS Public Access.

[59] Maibaum L., Dinner A.R.R., Chandler D. (2004). Micelle formation and the hydrophobic effect. J. Phys. Chem. B.

[60] Lang J., Zana R. (1986). Effect of alcohols and oils on the kinetics of micelle formation-breakdown in aqueous solutions of ionic surfactants. J. Phys. Chem..

[61] Celle C., Cabos A., Fontecave T., Laguitton B., Benayad A., Guettaz L., Pélissier N., Nguyen V.H., Bellet D., Muñoz-Rojas D., Simonato J.-P. (2018). Oxidation of copper nanowire based transparent electrodes in ambient conditions and their stabilization by encapsulation: Application to transparent film heaters. Nanotechnology.

[62] Manning H.G., Niosi F., da Rocha C.G., Bellew A.T., O’Callaghan C., Biswas S., Flowers P.F., Wiley B.J., Holmes J.D., Ferreira M.S. (2018). Emergence of winner-takes-all connectivity paths in random nanowire networks. Nat. Commun..

[63] Haacke G. (1976). New figure of merit for transparent conductors. J. Appl. Phys..

[64] Khaligh H.H., Xu L., Khosropour A., Madeira A., Romano M., Pradére C., Tréguer-Delapierre M., Servant L., Pope M.A., Goldthorpe I.A. (2017). The Joule heating problem in silver nanowire transparent electrodes. Nanotechnology.

[65] Khaligh H.H., Goldthorpe I.A. (2013). Failure of silver nanowire transparent electrodes under current flow. Nanoscale Res. Lett..

[66] Sannicolo T., Charvin N., Flandin L., Kraus S., Papanastasiou D.T., Celle C., Simonato J.-P., Muñoz-Rojas D., Jiménez C., Bellet D. (2018). Electrical mapping of silver nanowire networks: A versatile tool for imaging network homogeneity and degradation dynamics during failure. ACS Nano.

[67] Copinet A., Bertrand C., Govindin S., Coma V., Couturier Y. (2004). Effects of ultraviolet light (315 nm), temperature and relative humidity on the degradation of polylactic acid plastic films. Chemosphere.

[68] Rivaton A., Chambon S., Manceau M., Gardette J.L., Lemaître N., Guillerez S. (2010). Light-induced degradation of the active layer of polymer-based solar cells. Polym. Degrad. Stab..

[69] Gorham J.M., MacCuspie R.I., Klein K.L., Fairbrother D.H., Holbrook R.D. (2012). UV-induced photochemical transformations of citrate-capped silver nanoparticle suspensions. J. Nanoparticle Res..

[70] Herrling T., Jung K., Fuchs J. (2006). Measurements of UV-generated free radicals/reactive oxygen species (ROS) in skin. Spectrochim. Acta Part A Mol. Biomol. Spectrosc..

[71] Rittié L., Fisher G.J. (2002). UV-light-induced signal cascades and skin aging. Ageing Res. Rev..

[72] Takagi K., Nair S.V., Watanabe R., Seto K., Kobayashi T., Tokunaga E. (2017). Surface plasmon polariton resonance of gold, silver, and copper studied in the kretschmann geometry: Dependence on wavelength, angle of incidence, and film thickness. J. Phys. Soc. Jpn..

[73] Duan J.L., Cornelius T.W., Liu J., Karim S., Yao H.J., Picht O., Rauber M., Müller S., Neumann R. (2009). Surface plasmon resonances of Cu Nanowire Arrays. J. Phys. Chem. C.

[74] Seifert M., Vargas J.E.B., Bobinger M., Sachenhauser M., Cummings A.W., Roche S., Garrido J.A., Sachsenhauser M., Cummings A.W., Roche S. (2015). Role of grain boundaries in tailoring electronic properties of polycrystalline graphene by chemical functionalization. 2D Mater..

[75] Platzman I., Brener R. (2008). Oxidation of polycrystalline copper thin films at ambient conditions. J. Phys. Chem. C.

[76] Fleisch T.H., Mains G.J. (1982). Reduction of copper oxides by UV radiation and atomic hydrogen studied by XPS. Appl. Surf. Sci..

[77] Park J.-H., Natesan K. (1993). Oxidation of copper and electronic transport in copper oxides. Oxid. Met..

[78] Wan Y., Wang X., Sun H., Li Y., Zhang K., Wu Y. (2012). Corrosion behavior of copper at elevated temperature. Int. J. Electrochem. Sci..

[79] Lee S.-K., Hsu H.-C., Tuan W.-H. (2016). Oxidation behavior of copper at a temperature below 300 °C and the methodology for passivation. Mater. Res..

[80] Papadimitropoulos G., Vourdas N., Vamvakas V.E., Davazoglou D. (2005). Deposition and characterization of copper oxide thin films. J. Phys. Conf. Ser..

[81] Nerle U. (2013). Thermal oxidation of copper for favorable formation of cupric oxide (CuO) semiconductor. IOSR J. Appl. Phys..

[82] Ramanandan G.K.P., Ramakrishnan G., Planken P.C.M. (2012). Oxidation kinetics of nanoscale copper films studied by terahertz transmission spectroscopy. J. Appl. Phys..

[83] Deal B.E., Grove A.S. (1965). General relationship for the thermal oxidation of silicon. J. Appl. Phys..

[84] Mehrer H. (2007). Diffusion in Solids: Fundamentals, Methods, Materials, Diffusion-Controlled Processes.

[85] Won Y., Kim A., Yang W., Jeong S., Moon J. (2014). A highly stretchable, helical copper nanowire conductor exhibiting a stretchability of 700%. NPG Asia Mater..

[86] Berean K., Ou J.Z., Nour M., Latham K., McSweeney C., Paull D., Halim A., Kentish S., Doherty C.M., Hill A.J. (2014). The effect of crosslinking temperature on the permeability of PDMS membranes: Evidence of extraordinary CO_2_ and CH_4_ gas permeation. Sep. Purif. Technol..

[87] Dameron A., Davidson S., Burton B., Carcia P., McLean R., George S. (2008). Gas diffusion barriers on polymers using multilayers fabricated by Al_2_O_3_ and rapid SiO_2_ atomic layer deposition. J. Phys. Chem. C.

